# Comparative cardio and developmental toxicity induced by the popular medicinal extract of *Sutherlandia frutescens* (L.) R.Br. detected using a zebrafish Tuebingen embryo model

**DOI:** 10.1186/s12906-018-2303-9

**Published:** 2018-10-05

**Authors:** Longsheng Chen, Minjie Xu, Zhunan Gong, Samkele Zonyane, Shuwen Xu, Nokwanda P. Makunga

**Affiliations:** 1 Anhui Academy of Applied Technology, Suixi Road 312, 230031 Hefei, Anhui People’s Republic of China; 2 Center for New Drug Research and Development, College of Life Science, Nanjing Normal University, No 1 Wenyuan Road, Nanjing, 210046 China; 3 Department of Botany and Zoology, Stellenbosch University, Private Bag X1, Matieland, 7600 South Africa

**Keywords:** Aqueous and ethanol extract, Cardiotoxicity, Cycloartane glycosides, Cytotoxicity, In vivo model, *Lessertia*, Medicinal plants, Plant metabolomics, Teratogenicity, Terpenoids

## Abstract

**Background:**

*Sutherlandia frutescens* is one of the most promising commercialized, indigenous and medicinal plants of South Africa that is used as an immune-booster, and a traditional treatment for cancer. However, few studies report on its toxicology and dosage in vivo. There is still room to better understand its cytotoxicity effects in animal systems.

**Methods:**

We prepared two extracts, one with 80% (*v*/v) ethanol, and the other, with water. Both were studied to determine the maximum tolerable concentration when extracts were applied at 0 to 200 μg/ml to a Tuebingen zebrafish embryo line. The development of zebrafish embryos after 24 h post fertilization (hpf) was studied. A concentration range of 5 μg/ml to 50 μg/ml was then chosen to monitor the ontological development of cultured embryos. A liquid chromatography-mass spectrometry/mass spectrometry (LC-MS/MS) method was used to study the differences of the two experimental extracts. Chemical variation between the extracts was illustrated using chemometrics.

**Results:**

Both extracts led to bleeding and pericardial cyst formation when applied at high concentrations to the zebrafish embryo culture. Chronic teratogenic toxicities, leading to pericardial edema, yolk sac swelling, and other abnormal developmental characteristics, were detected. The aqueous extracts of *S. frutescens* were less toxic to the larvae than the ethanol extracts, validating preference for aqueous preparations when used in traditional medicine. Chemical differences between the water extracts and alcoholic extracts were analysed using LC-MS/MS. A supervised metabolomics approach, targeting the sutherlandiosides and sutherlandins using orthogonal partial least squares-discriminant analysis (OPLS-DA), illustrated that sutherlandiosides were the main chemical features that can be used to distinguish between the two extracts, despite the extracts being highly similar in their chemical constituents.

**Conclusion:**

The water extract caused less cytotoxic and abnormal developmental effects compared to the ethanolic extract, and, this is likely due to differences in concentrations of extracted chemicals rather than the chemical profile per se. This study provides more evidence of cytotoxicity effects linked to *S. frutescens* using the zebrafish embryo bioassay as a study tool.

**Electronic supplementary material:**

The online version of this article (10.1186/s12906-018-2303-9) contains supplementary material, which is available to authorized users.

## Background

*Sutherlandia frutescens* (L.)R.Br. (also taxonomically referred to as *Lessertia frutescens*) belongs to the legume family (Fabaceae) and is an important traditional medicinal plant in South Africa that has commercial value. It is widely distributed in South Africa’s Western Cape region. Wild growing plants are also found in certain parts of Mpumalanga and KwaZulu-Natal provinces. The southern African countries of Botswana and Namibia [1] also have populations of this particular species. The plant is a small, soft shrub with a height of about 0.5 to 1 m. It has grey-green pinnate leaves that are about 4–10 mm long. It is characterised by a bitter taste and orange-red flowers that are about 35 mm long and these flowers generally appear annually during spring to mid-summer and this spans the time period of September to January in the Southern hemisphere. This species is used to generate a suite of phytopharmaceutical products which are sold as stress-relieving immunotonics [2]. Many consumers may purchase products which are sold over the counter, made of the dried plant material collected from the foliage or extracted tinctures, to take them for relieving anxiety over-extended periods of time. These plants have been shown to possess anti-inflammatory bioactivity [3] and anti-diabetic effects [4]. Various commercial herbal products that target Type 2 diabetic patients and those people suffering from anxiety are made from the leaves of the plant. The dried leaves are used to produce tinctures, teas and capsules which are sold in the complementary and alternative medicines sector in South Africa and abroad [2].

*S. frutescens* has a long history of use as a traditional medicine and has been widely used in the treatment of many kinds of human diseases such as asthma, dysentery, fever, gastritis, diabetes; and in folklore, it is a reputed treatment for cancer [5]; hence, its common name of cancer bush. Because of this reputation, it is often included in plant polyherbal remedies that are administered by traditional herbal healers [6] and they claim that, it is powerful in especially ‘boosting the immune system in order to fight disease’. Recent studies focusing on its cytotoxicity effects include the work of Skerman et al. [7], Vorster et al. [8], Mqoco et al. [9] and Leisching et al. [10] using various anti-cancer cell lines. Thus far, there are no studies that illustrate the effect of *Sutherlandia*-derived extracts using a zebrafish model.

The zebrafish, also known as *Danio rerio*, is a model in drug screening assays that are also promising for studying human diseases [11]. An increasing number of academic institutions and pharmaceutical companies have recognized the use of monitoring zebrafish embryo development, over the past decade, as a powerful tool in drug discovery, which has potential to speed up screening processes of new therapeutic drugs for humans [12]. As a research subject, its application possesses many critical advantages over other traditional vertebrate models [13, 14] because of: 1) the rapid generation time for embryo development; 2) high fecundity; 3) external embryonic development; 4) a capacity for high stocking density in relatively small areas; and, 5) lower maintenance costs [15]. These features, amongst others, are used as markers to determine the extent of cytotoxicity to various chemical agents and solutions as part of toxicological studies during the drug discovery process for allopathic and natural medicines. Several developmental abnormalities are expressed in zebrafish that are characterised by pericardial and yolk sac oedema, excessive curvature of the spine, heart malformation, disfigured lower jaw growth, and disturbances to cardiovascular circulation [15]. Various extracts from different plant species have thus been studied using zebrafish (for details refer to Atanosov et al. [16]). This bioassay is particularly attractive for studying plant extracts used for human health because zebrafish share physiological, morphological and genetic homology to many other higher vertebrate systems [17].

Studying plant-derived extracts is generally challenging as different extraction solvents, extraction methods or various storage conditions give rise to different plant active ingredients, leading to variation in the mode of action associated with plant extracts. The influence is largely pertaining to their pharmacodynamics and pharmacological action. So far, water, methanol, ethanol, chloroform, dichloromethane, acetone, hexane and so on, are popular extraction solvents, and, a ton of literature indicates the efficacy of these different extracts. A water decoction is one of the most extensively studied preparations of *S. frutscens* as this is the general way in which an extract is made in traditional medicine. Even so, no extracts of *Sutherlandia* have been studied using the zebrafish bioassay. Recently, several studies have shown the presence of sutherlandins and sutherlandiosides as key ingredients that are uniquely synthesised by *S. frutescens* [1]. These are now used as biomarker molecules to monitor the quality of plant material that is either harvested from the wild or produced in field cultivation for the manufacturing of a variety of naturopathic products derived from this plant [1]. Although the active principles responsible for various biological activities of *S. frutescens* extracts remains unresolved, several authors have hinted that flavonoids and terpenoids may be likely candidates that possess bioactivity [18], as these plants manufacture very specialised metabolites, sutherlandins and sutherlandiosides, that belong to these chemical groups, respectively. Our interest was geared to further understanding of the toxicity of *S. frutescens*, especially linked to possible cardiotoxicity and developmental toxicity, hence the application of extracts to developing zebrafish embryos.

Therefore, the aim of this article was to explore the possible toxicity of two extracts of *S. frutescens.* One of these was prepared using ethanol whilst the other was produced using water, termed here: S.fru-OH and S.fru-H_2_O extracts, respectively. By determining the hatching rate, mortality, morpho-physiological changes, cardiotoxicity effects and behavioural aspects linked to zebrafish embryos and their ontogenetic development, we could better elucidate cytotoxicity effects associated with both extracts. This work also includes an assessment of the metabolomic profile of each extract using liquid chromatography-mass spectrometry (LC-MS) where sutherlandins and sutherlandiosides could be monitored easily. These are flavonoids and terpenoids specifically associated with *S. frutescen*s, and so, the chemical fingerprints of the two extracts were also studied.

## Methods

### Plant collection

*Sutherlandia frutescens* plants were collected from wild populations within the Karoo region, a semi-desert area which is known to have low rainfall and high temperatures in summer. Plants were collected from various localities (31°02′26”S 25°44′08″E; 31°4′6”S 24°26′23″E; 30°58′32”S 24°37′22″E and 30°29′49”S 27°09′71″E) when they were in flower to allow ease of identification, as they display orange-red flowers during this time (November 11, 2014, to November 23, 2014). Voucher specimens (SF (Z14); SF (Z15); SF (Z16)) were lodged at Stellenbosch University’s Herbarium in the Department of Botany and Zoology. Each voucher specimen is accompanied by dried floral parts, collected from representative individuals. At the time of collection, botanical identification was conducted by Samkele Zonyane (SZ). Other features that were used to identify the plants included bladder-shaped paper seed pods and petiolate green to grey leaves. Collection of plant material was possible as permits were issued by CapeNature (00280AAA008-AA165) and the Department of Economic and Environmental Affairs (CRO 104/14/CR; CRO 105/14CR) to SZ.

Plants from four locations were pooled together and collected to form a 1 kg batch of air-dried material (at room temperature), as this is a common practice of commercial manufacturers that make *Sutherlandia* products. The plant material was then ground to a fine powder using a Breville mill before being passed through a 500 μm pore mesh sieve to obtain material of a uniform particle size. The plant material was stored dry, in the dark, and, at room temperature until further use.

### Phytochemical extraction

We were interested in testing two types of extracts and so one was made with ethanol and the other was made with water. For each extraction, 100 g powder was soaked in distilled water or in a solution of 80% (*v*/v) ethanol prior to ultrasonication (KH-100DE numerical control ultrasonic cleaners) for 30 min. The water extract was then assigned as S.fru-H_2_O; and, the ethanolic extract was termed the S.fru-OH extract. Each extract was then filtered using Whatman No1 paper. The extraction was repeated twice on the same material with ultrasonic extraction, lasting a period of 30 min each time. The respective extracts were then pooled together before the water extract (S. fru-H_2_O) and the 80% ethanol extract (S. fru-OH) were reduced in vacuo. Twenty milligrams of each dried extract was redissolved with either distilled water or 80% (*v*/v) ethanol (respectively) and made to a 2 mg /mL stock. Thereafter, the extracts were filtered using a 0.22 μm sterile filter into 10 mL volumetric flasks and stored at 4 °C when not in use. These solutions were then used in the zebrafish bioassay.

### Zebrafish bioassay

#### Animal culture conditions and housing

The adult wild-type zebrafish (Tuebingen line) was provided by the Model Animal Research Center of Nanjing Normal University for this study. The animals were incubated in charcoal-filtered oxygenated tap water, grown using a 14:10 h light/dark cycle at a temperature of 28.5 °C and the pH was adjusted to 7 ± 0.2 [19]. To maintain the animals in culture, they were fed with live brine shrimp once daily and dry food twice a day [20]. The animals are sexually mature at about 3 months and they have a survival time of about 2 years. The culture medium used was the zebrafish embryo culture medium of Lia et al. [21] which was supplemented with an antifungal agent (0.01% (*v*/v) methylene blue) and salts (5 mM NaCl, 0.17 mM KCl, 0.33 mM CaCl_2_ and 0.33 mM MgSO_4_).

#### Ethical statement

All zebrafish studies were approved by the Institutional Animal Care and Use Committee at Nanjing Normal University. Ethical clearance for the study was obtained by author ZG (permit number: 2090658) and the work was conducted in his laboratories according to institutional provisions.

#### Zebrafish embryo collection

Zebrafish embryos were obtained from spawning adults in tanks overnight with a 1:2 male to female (*v*/v) ratio. Pairwise mating in a fish hatch box was utilized to produce embryos, and these were then collected within an hour post fertilization before being washed 3 times, and, then raised under illumination in an incubator at 28 °C. Normally, developed embryos were observed every day and dead embryos were counted and removed from the tanks daily. Every 24 h, fresh solutions were made to replace those in growth tanks. For all experiments that followed, a random design was utilized to collect animals.

#### The determination of exposure concentrations

For the first experiment, performed in 2016, embryos (larvae) were exposed to the S.fru-OH and S.fru-H_2_O extracts. When these extracts were compared, there were no signs of dead or abnormality of embryos (larvae) within 8 h post fertilization (hpf) when a concentration range of 5 μg/ml to 50 μg/ml was used. This range was determined to be the maximum tolerable concentration (MTC) [22]. However, the larvae were all dead after 24 h, when the exposure concentration ranged between 100 to 200 μg/ml. Therefore, the exposure concentration that was used throughout this study was confirmed to be 5 μg/ml - 50 μg/ml. A set of concentrations from the lowest to the highest were then tested. Controls were left untreated.

#### Treatment of control and experimental groups

**Fig. 1:**
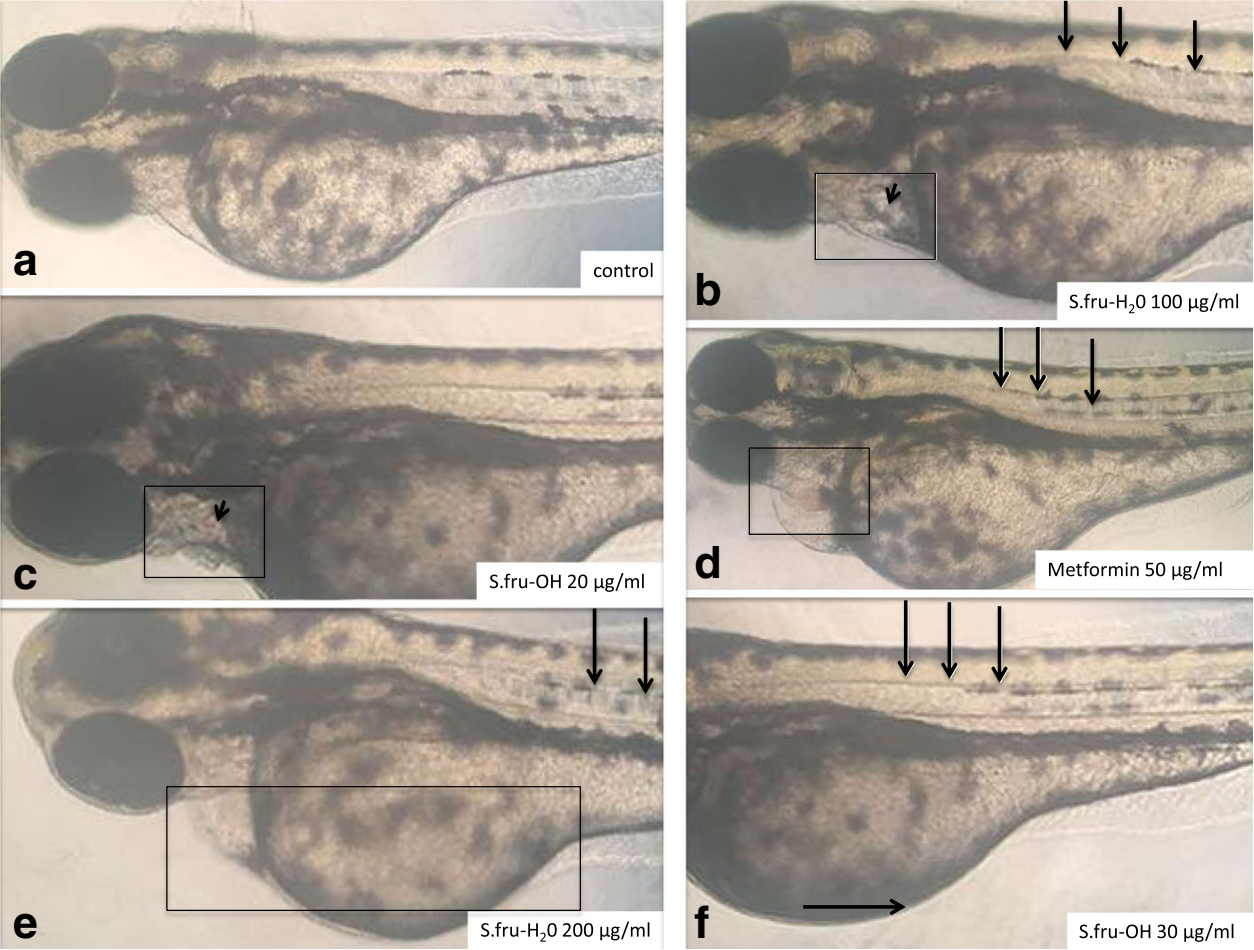
Embryos treated with different concentrations of S.fru-OH or S.fru-H_2_O at 96 hpf. **a** An example of a control (untreated) embryo with a normally developed straight spine without any visible signs of bleeding. **b** The short arrow points to a darkened region where a cyst is forming. The boxed zone highlights the pericardial region. **c** The short arrow points to a pink discolouration within the pericardial zone (shown using a rectangle). **d** A distinct pink sphere is visible within the pericardial zone (rectangle) and long arrows point to a curving spine. **e** A clear mass of tissue and an enlarged abdominal region is highlighted by the rectangle. **f** Long arrows point to a slightly curving spine. Pericardial cyst formation caused by exposure of zebrafish embryos to S.fru-H_2_O at 100 and 200 μg/ml was also accompanied by enlarged bellies (shown here by a horizontal arrow)

The preliminary organs of zebrafish embryos and their ability to function is virtually fully formed and mature at 96 hpf [23]. At this time point, larvae were hatched from the chorion and so, we choose 96 hpf point to do the toxicity experiment and observe the changes in morphology of zebrafish [23], refer to Fig. 1. To aid with the visual identification of abnormalities in developing zebrafish embryos, metaformin (an anti-diabetic drug) was applied to a set of embryos as a positive control at a concentration of 50 μg/ml. The embryos with a survival rate of over 95% were chosen for this particular set of experiments. After microscopic examination according to the work of Parng et al. [24], normal embryos at 24 hpf were randomly distributed into a 24-well plate and 6 embryos were placed per well. The control group was exposed to embryo culture medium only, while the experimental group was exposed to S.fru-OH and S.fru-H_2_O extracts, respectively, at different concentrations. Each embryo was regarded as a replicate. For each treatment, triplicates were used for experimentation. All test embryos were then grown at 28 ± 0.5 °C using a 12 h light: 12 h dark cycle [22]. Data that were collected were linked to the heartbeats, pericardial cyst formation, autokinetic movement, malformation stress response, death and/or survival numbers of the zebrafish [25]. Measurements were recorded every day during the experiment. In addition to that, the mortality rate and hatching rate were calculated after exposure to the botanical extracts.

#### Metabolomic analysis of extracts

Liquid chromatography-mass spectrometry was performed using a Waters Acquity ultra-performance liquid chromatography (Milford, MA, USA) with an Acquity photodiode array (PDA) detector and an autosampler that is coupled to a Waters Synapt G2 quadrupole time-of-flight mass spectrometry. One microlitre of both extracts was analysed for the respective replicates using a Waters UPLC BEH C18 column (2.1 mm × 100 mm, 1.7 μm particle size). Solvent A, made up of 0.1% (*v*/v) formic acid, and Solvent B, using acetonitrile, were combined to generate a mobile phase as follows: 95% A: 5% B (0–5 min), 56% A: 44% B (6–20 min), 0% A: 100% B (20–22 min), 95% A: 5% B (22–26 min) at a flow rate of 350 μL/min. The total run time for each sample was 26 min. A cone voltage of 15 V (positive ionisation mode) and a capillary voltage of 3.0 kV was set and helium was used as the desolvation gas (650 l/h flow rate) at a temperature of 275 °C. The LC-MS/MS method used here was similar to that developed by Albrecht et al. [1] and used also by Grobbelaar et al. [26].

To compare the metabolite profiles of the two extracts, the MassLynx 4.1 software program, using the TargetLynx as an application manager, was used. The sutherlandins elute between 7 to 9 min and the sutherlandiosides are visible at a retention time of 15 to 19 min. Retention times, mass spectra (MS), UV spectra and the fragmentation patterns generated through tandem mass spectrometry (MS/MS) were used to identify *Sutherlandia*-specific compounds. Spectra were aligned to determine regions of similarity that may be responsible for observed effects on the zebrafish model when comparing the water and the ethanol extracts. A principal component analysis (PCA-X model) was performed as an unsupervised pattern recognition approach and this utilises pareto-scaling as a means to differentiate chemical profiles based on compound differences. For this, a level of 1000 was set for noise elimination and smoothing. Due to high similarity of the metabolite profiles of both extracts, PCA analysis was followed by partial least squares discriminant analysis (PLS-DA) as a supervised technique which assisted with dimension reduction as the PCA was not resolving well. Although PLS-DA is known for overfitting, it assisted to reduce the influence of noisy variables, clarifying the data set so that we were able to detect those compounds that were able to separate the two extracts. Data analysis was based on MS data, relative abundances, spatial distribution and scores plots highlight outliers that distinguished the samples that are outside the model boundary linked to Hotelling’s T ellipse. As a chemical standard, sutherlandioside B was used (Additional file 1).

#### Analysis of sutherlandioside B

Quantification of sutherlandioside B was possible using a chemical reference standard where a calibration curve, linear in the range of 0 to 50 ppm (R^2^ = 0.998), was used and for the confirmation of the peak using Masslynx version 4.1. The detection limit of 0.01 ppm was established and the relative standard deviation was less than 3% in terms of day-to-day precision and within 1 day of analysis (*n* = 6/day for replicate injections). For this method, five replicate injections were done for reproducibility and the relative standard deviation of less than 3% was recorded. This work was conducted at the Central Analytical Facility of Stellenbosch University and this technique is also reported by Grobbelaar et al. [26] and the LC-MS/MS method is used routinely in our environment.

### Statistical analysis

#### Univariate statistics

For the zebrafish bioassay, statistical analysis was conducted in triplicate and all the values are expressed as mean ± standard error (SE) or mean ± standard deviation (SD). The LC_50_ was calculated using SPSS Statistics. One way analysis of variance (ANOVA) and Dunnett’s multiple comparison tests were performed to determine the significant differences (*P* < 0.05) between the means by using Graphpad Prism 6.0.

#### Multivariate statistics

For the metabolomic data, PCA and the OPLS-DA were conducted for both sets of extracts. The PCA gave poorer resolution and this was then followed with a supervised analysis using the OPLS-DA to reduce the dimensionality of the data.

## Results

### Morphological effects on zebrafish embryo development

The intensity of phenotypic deformities induced by *Sutherlandia* extracts is shown in Fig. 1. As shown in Fig. 1, concentrations of 0–5 μg/ml for both the S.fru-OH and S.fru-H_2_O extracts had no deleterious effects on the development of zebrafish (refer specially to Fig. 1a for an example of normal development). We were able to identify some of the features that are associated with abnormal development of zebrafish [27] such as enlarged and dense tissue growth with various treatments; and pink discolouration (Fig. 1b-d) of tissues due to bleeding.

**Fig. 2:**
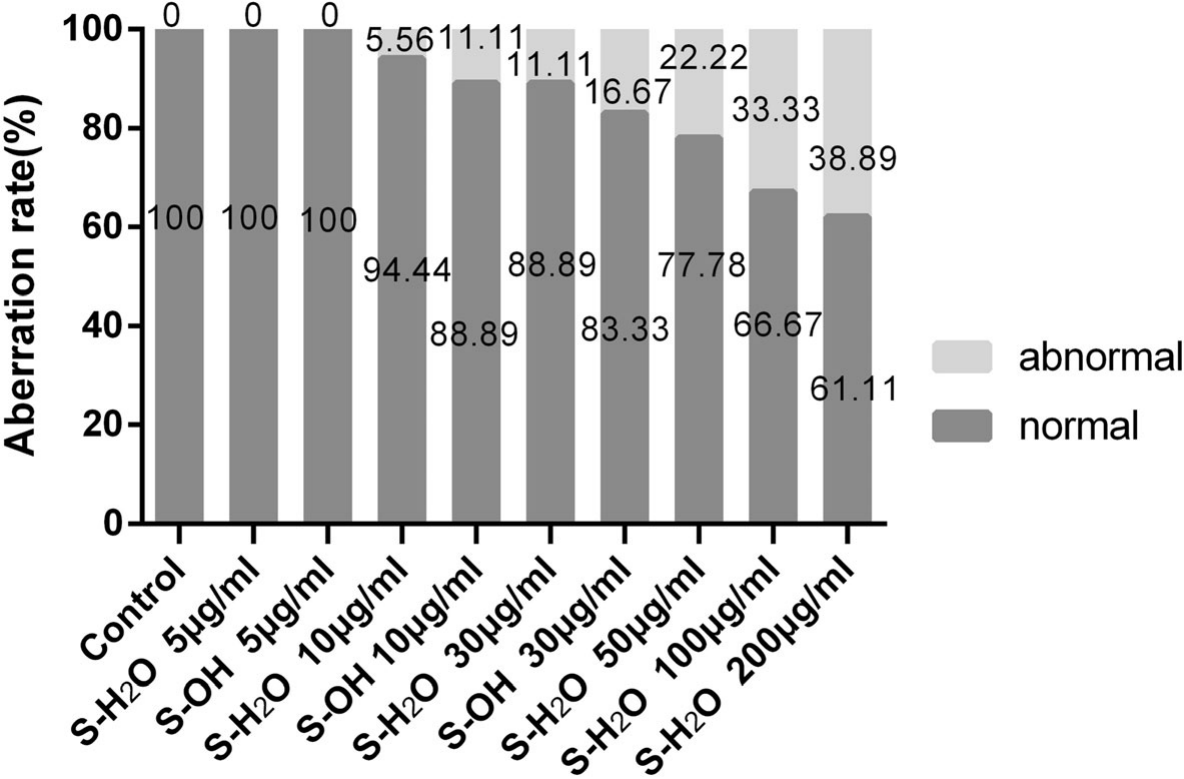
The percentage of morphologically abnormal and normal zebrafish treated with different concentrations of S.fru-OH or S.fru-H_2_O compared to the control (*n* = 18 per group)

With increasing concentrations of the S.fru-OH and S.fru-H_2_O extracts, embryos progressively showed signs of poor development. For instance, those exposed to the S.fru-OH, at 30 μg/ml, exhibited abdominal excess fluid and yolk sac oedema. The same situation was observed when the concentration of the S.fru-H_2_O extract was raised to 100 μg/ml and above. For example, an embryo with an enlarged yolk sac and a curving spine (treated with 200 μg/ml S.fru-H_2_O extract) is shown in Fig. 1e. Such embryos exhibited poor mobility. The first signs of spinal curvature for those zebrafish embryos exposed to the ethanol extract was evident even when embryos were exposed to extracts of a concentration of 30 μg/ml (Fig. 1f). Data presented in Fig. 2 further confirm that the toxicity of S.fru-OH to zebrafish is greater than the S.fru-H_2_O. Higher doses of the ethanolic extract were linked to greater incidence of aberrant morphological formations, recorded at a frequency of 38% when embryos were exposed to a 200 μg/ml extract (Fig. 2).

### Cardiotoxicity effects

**Fig. 3:**
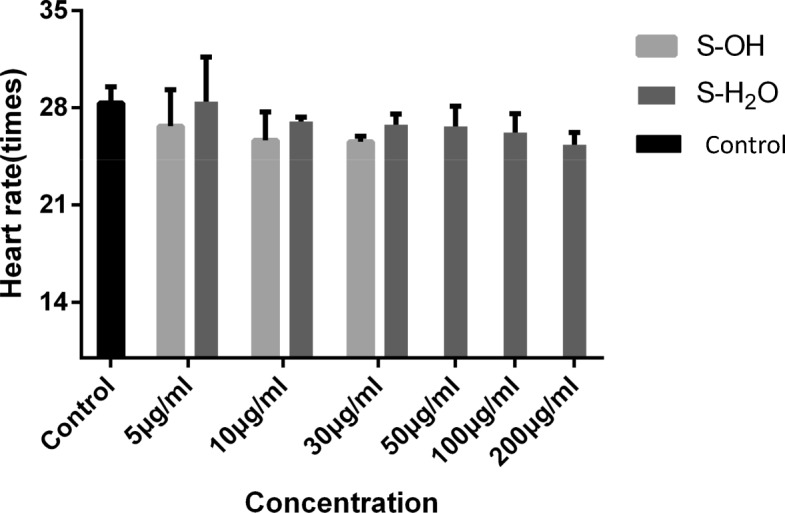
Cardiotoxicity in zebrafish embryos caused by different concentrations of S.fru-OH (5, 10 and 30 μg/ml) or S.fru-H_2_O (5, 10, 30, 50, 100 and 200 μg/ml). The heart rate was measured at 96 hpf and the values are described as mean ± SD

Heartbeats were recorded at 96 hpf to determine the protective effect of the S.fru-OH or S.-fru-H_2_O on cardiac function against cardiotoxicity (Fig. 3). The average heart rate in the control group was 28 ± 1 beats/10s while a significant decrease (27 ± 3 beats/10s) in heart rate was observed in embryos exposed to the S.fru-OH plant extract at 5 μg/ml. However, the S.-fru-H_2_O group did not show significant decreases in the measured heart rate as these were counted at 28 ± 3 beats/10s. This value was very close to the control group which was not exposed to the phytoextract. On the other hand, embryos exposed to the S.fru-OH (10 μg/ml and 30 μg/ml) had lowered heartbeats than control embryos. This phenomenon of decreasing heartbeats was less evident when embryos were exposed to the S.fru-H_2_O extract (10 to 200 μg/ml). When the highest concentration of the water extract was applied to the test larvae at 100 and 200 μg/ml, beats of 26 ± 1 beats/10s and 25 ± 1 beats/10s were recorded (respectively). In some embryos, we noticed signs of bleeding and/or small pericardial cysts appeared on the zebrafish embryos when the concentration of S.fru-H_2_O was raised to100 μg/ml and above (Fig. 1b).

### Zebrafish mortality and hatching rates

**Fig. 4:**
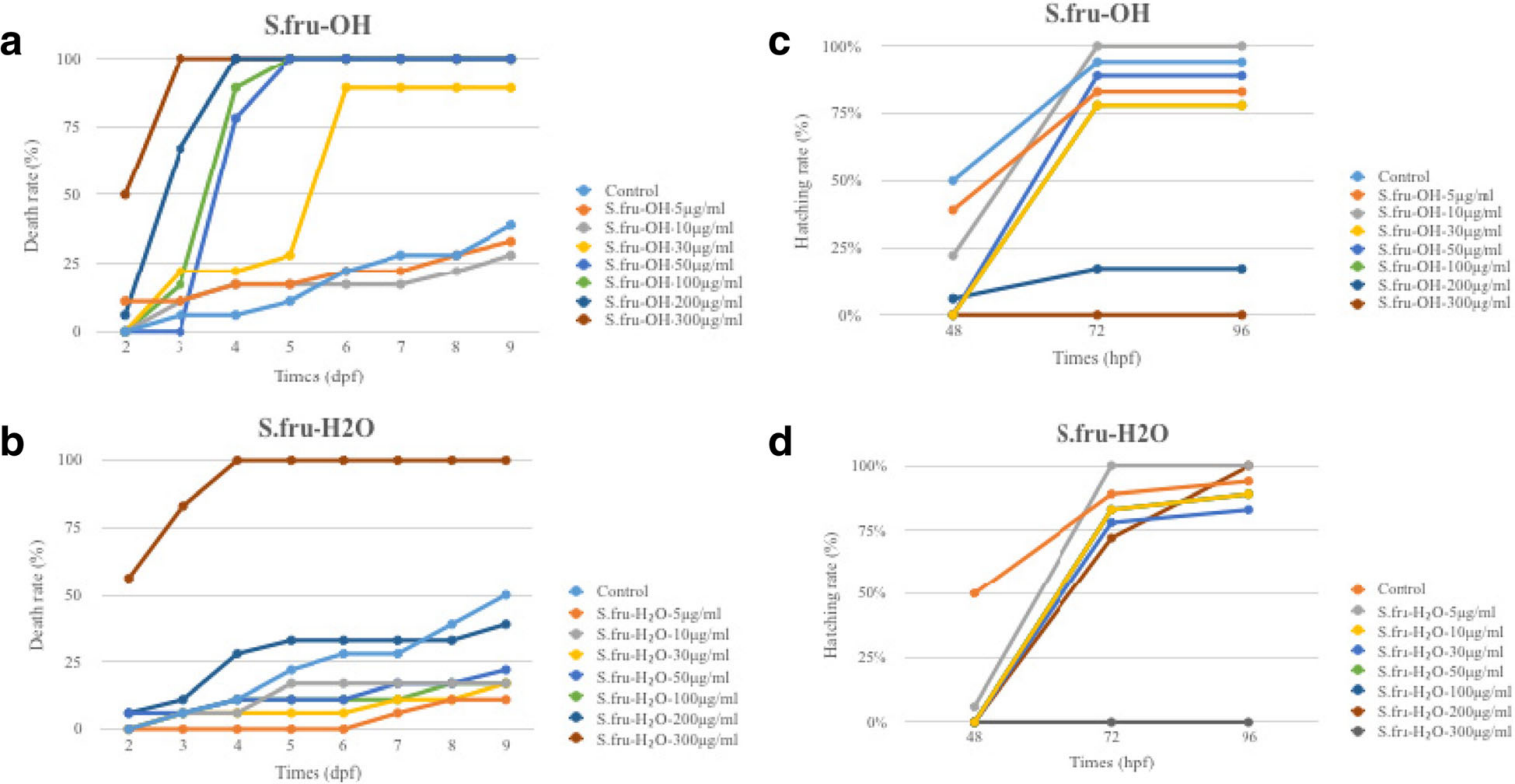
The death rate of zebrafish embryos exposed to various concentrations of *S. frutescens*. **a** S.fru-OH extract and **b** S.fru-H_2_O were applied at 0–300 μg/ml; and, the hatching rate of zebrafish embryos exposed to various concentrations of *S. frutescens*. **c** S.fru-OH extract and **d** S.fru-H_2_O were applied at 0–300 μg/ml

A treatment of 300 μg/ml with both extracts, which was the highest concentration tested in this study, resulted in acute lethal toxicity for the S.fru-OH extracts (Fig. 4a). At this particular concentration, the S.fru-OH was fatal for the test animals, with virtually all the embryos being unable to survive on the third day. A similar trend was observed for the water extract when it was applied at the highest concentration tested. When the concentrations were lower than 30 μg/ml, the mortality rate increased gradually and was correlated to the number of days that the zebrafish embryos were exposed to the extract (Fig. 4a). At day 9, the only surviving embryos were those exposed to the 5 μg/ml and 10 μg/ml plant extract together with the controls. After 4 dpf, the death rate of each group for the S.fru-OH treatment rapidly increased, and this was especially prevalent with those concentration groups (50, 100, 200 and 300 μg/ml) which are over the 30 μg/ml range. These treatments induced a death rate of 78, 89 and 100%, plus, all animals had died at 4 dpf (Fig. 4a-b). Overall, the high mortality was associated with the S.fru-OH applied at concentrations higher than 100 μg/ml whereas the S.fru-H_2_O group was able to survive even when applications of 100 μg/ml were used. Embryos continued growing and developing until the termination of the experiment at dpf 9. The effects of S.fru-OH and S.fru-H2O on the hatching rate of zebrafish embryos is shown in Fig. 4c-d. No zebrafish embryos had hatched when the dose of the treatment was 300 μg/ml, irrespective of extraction method used to generate the herbal drug. Use of the 200 μg/ml S.fru-OH extract also negatively affected the hatching rate, leading to less than 25% of the embryos that had hatched. The hatching rate of the control group was recorded to be 50% at 48 hpf, which was higher than the drug treatment groups irrespective of whether the S.fru-OH or S.fru-H_2_O extract was being examined (Fig. 4c-d). But, when exposure time was over 48 hpf, the hatching rate of each of the groups started to increase and recorded numbers for the hatching frequency were very close to the control group. These trends suggested that the S.fru-OH, especially at high concentrations, had toxic effects which are responsible for delaying hatching. As expected, lower doses of S.fru-OH (5 or 10 μg/ml), generated a less pronounced delayed hatching effect compared to when higher doses of this extract were applied (Fig. 4d).

### Chemical profiling of the extracts

**Fig. 5:**
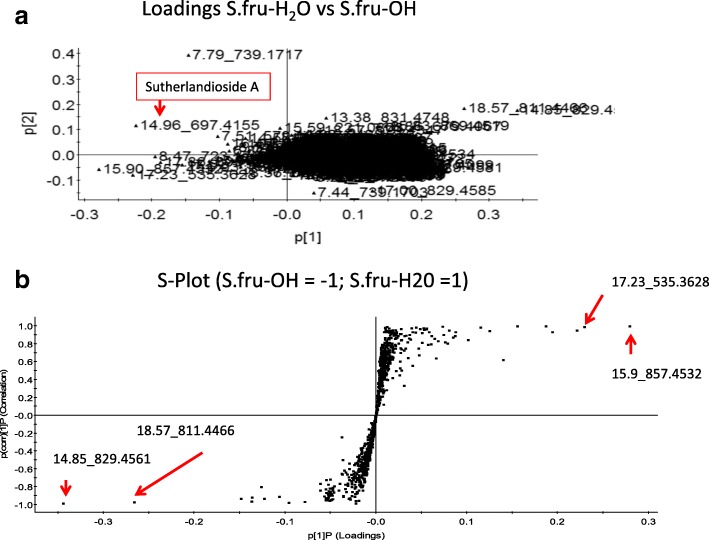
**a** Loadings plots based on PCA separation of S.fru-OH extract and S.fru-H_2_O. **b** S-Plot of the PLS-DA method used to study the S.fru-OH [= − 1] and S.fru-H_2_O [=1] extracts of *S. frutescens*

In this study, the PCA showed the presence of many chemicals that were shared in the two extracts as expected (Fig. 5a) but the two extracts could be separated from each other. The chemical sutherlandioside A was significant in this separation, occurring in the bottom left quadrant of the PCA scores plot. The loadings from a two-class OPLS-DA model, comparing group 1 vs. group 2 representing the S.fru-OH and S.fru-H2O extracts respectively, are shown in the S-plot format (Fig. 5b). The points are exact mass/retention time pairs (EMRTs) plotted by covariance (x-axis) and correlation (y-axis) values. The upper right quadrant of the S-plot shows those components which are elevated in the S.fru-H_2_O extract, while the lower left quadrant shows components elevated in the S.fru-OH group. We used several sources from the primary literature to identify several chemicals that are known to occur in *S. frutescens* extracts (for details refer to Albrecht et al. [1], Grobbelaar et al. [26]; Acharya et al. [28]) and Additional file 1 shows the chromatograms of the test samples. Sutherlandioside C (m/z 695.3996 [M + formate]^−^ eluting at 17.86 min) was detected in the aqueous extract. Sutherlandioside A with a base peak of 697.4155 [M + formate]^−^ was identifiable (Fig. 5) and this chemical eluted at 14.96 min. Histograms represent the most important chemicals (or EMRTs) associated with groupings (Additional file 2). We used LC-MS/MS (together with a reference standard) to confirm and quantify the levels of sutherlandioside B. The mass spectral ionisation pattern was useful in verifying the structural integrity of this chemical and five aglycone fragments were detected, namely, 491.3733 [agly+H]+, 473.3630 [agly+H-H2O]+, 455.3523 [agly+H-2H2O]+, 437.3411 [agly+H-3H2O] + and 419.3310 [agly+H-4H2O]. Water extracts had higher levels of sutherlandioside B at 0.033 μg / g whereas the S.fru-OH extract had a value of 0.0089136 5 μg / g dry weight of this chemical (data not shown).

## Discussion

### Zebrafish bioassay

To our knowledge, this is the first study to design and evaluate the teratogenic effects of *S. frutescens* extracts using a zebrafish bioassay approach. To facilitate this, our first step was to determine appropriate dosages for the application of the extracts to fertilized and developing fish embryos. Impacts at the embryogenic phases show developmental defects correlated to concentrations and extract type (Figs. 1 and 2). In general, abnormal development of zebrafish manifests through symptoms of oedema or bleeding under or around the abdomen and is also accompanied by body deformities, such as curvature of the spine and tail, development without a pectoral fin, pigmentation and enlarged yolk sacs with embryos having lowered mobility [27]. Teratogenicity recorded in zebrafish is critical as it reflects the predictive power of the bioassay for assessing developmental toxicity in mammals [27]. Studying these extracts showed that zebrafish development is acutely affected by high concentrations of both extracts and these are not only lethal to larvae but also, produce chronic teratogenic toxicity such as pericardial oedema, yolk sac swelling, bleeding and other characteristics deleterious to development and organ formation. We noted that the aqueous extract (S.fru-H2O) had a weaker toxic effect than the ethanol extract.

Normal function of the heart is essential for growth and development in later stages of life as poor heart function can cause severe developmental effects [15]. The observations associated with a decreasing heart rate when higher concentrations of the extract were supplied to the zebrafish (Fig. 1b) suggested that the S.fru-OH and S.fru-H_2_O would cause dose-dependent cardiotoxicity effects, which negatively affect the heart rate as it decreases. This is further exacerbated by the induction of pericardial cyst formation in zebrafish embryos. The heart of embryos is the organ that first develops and is necessary for the healthy functions of all other organs [15]. Proper function of the heart plays a key role for the normal development of the embryo in subsequent phases that follow. In other words, when the cardiac system is poorly developed, it may lead to abnormal general development of the animal causing severe malformations and poor organ functioning [15]. Moreover, evidence of the dull stress reaction and poor abdominal motor ability [12, 27] were further visual signs of the ill effects (data not shown) of these respective concentrations for both extracts studied in this paper. A prolonged exposure period to the phytoextracts, along with higher dosages of both the S.fru-OH and S.fru-H_2_O extracts in developing embryos, led to cardiac contractility, poor cardiac output and a heart rate that progressively slowed down. In cases where severe pericardial cyst formation occurs, it may slow down blood circulation and cause an inhibited heartbeat. This has profound implications as overexposure and overdose of *S. frutescens* derived extracts could cause toxicity of the heart in other mammalian organisms; and especially humans, who are using these botanical drugs for health purposes. The death of zebrafish when exposed to high concentrations of both extracts (Fig. 4a) may thus be related to severe cardiac malfunction. The higher concentrations of the extracts on the larvae resulted in acute lethal toxicity, while chronic teratogenicity was caused by exposing the fertilised embryos to lower concentrations (Fig. 4a). The hatching rates are shown in Fig. 4a and it is one of the most important indicators of toxicity evaluation when utilising a zebrafish model bioassay. Under normal circumstances, zebrafish embryos begin to hatch from the 48 hpf [19, 23]. Generally, most embryos hatch at 72 hpf and incubation is almost complete at 96 hpf. High rates of hatched embryos further confirmed that the S. fru-H_2_O is less harmful to zebrafish embryos. Clearly illustrated here, is that the hatching rate of the phytochemically-treated groups were lower than the control group at 48 hpf. We speculated that low doses of the extracts could play a role of delaying hatching by penetrating the vitelline membrane.

Phulukdaree et al. [29] studied a water extract of *S. frutescens* made from commercially available tablets and their data also suggested this type of extract is not cytotoxic when administered at low concentrations but high doses elicit oxidative stress that is accompanied by alterations to mitochondrial membranes. They further observed apoptosis in renal tubule epithelia and damage was more prominent when concentrations were high. This study also serves to confirm the cytotoxic effects of extracts of *S. frutescens* observed by others using various cancer cell lines. Although there is a growing body of evidence attesting to the biological action of *Sutherlandia* against various cancers [7–10], perceived cytotoxicity in this study also alludes to the precautions that need to be taken by users of these extracts especially when *S. frutescens* products are consumed routinely for health purposes.

### Metabolomic profiles

To better understand the chemistry of the two extracts, LC-MS/MS analysis was conducted and followed with a principal component analysis (PCA). Acharya et al. [28] utilized a similar chemometric study to analyse different populations of *S. frutescens*. Although there were many similarities between the water and the ethanol extracts based on their chemical features (Fig. 5), we were able to show that the ethanol extract had some chemicals that were distinct from the water extract (Additional files 1 and 2). Sutherlandioside A was important in differential clustering observed in this study. The separation of the water extract from the ethanolic-based group may provide one reason for the differences in cytotoxicity in the two extracts. Quantitative differences in terms of the biochemicals responsible for differentiating between these extracts may also explain why cytotoxicity is variable with the ethanol being more potent. Several sutherlandioside derivatives which eluted between 16 and 18.5 min accumulated in both extracts. Although differences in the flavonoids and terpenoids are not necessarily prominent between these two extracts, the relative abundance of these chemicals is likely to have a great impact on their toxicology (Fig. 4b; Additional file 2), contributing to differences displayed in bioactivity in the zebrafish bioassay. It thus remains important to identify the biochemicals responsible for bioactivity of *Sutherlandia* plant extracts. At this time, the chemistry has not yet been comprehensively studied, with only eight molecules being used as reporter chemicals [26, 28].

At this stage, a phytoextract with a complex mixture of many different biochemicals is the way in which *S. frutescens* products are consumed. There are currently no pure compounds isolated from this plant or synthesised in vitro in laboratories that are being sold as pharmaceutics. Once commercialisation of single pure compounds, such as sutherlandioside B occurs for human consumption, it will become more relevant to test individual compounds also for cytotoxicity. Application of the zebrafish bioassay in this regard may be important. So far, our work has shown the first source of evidence regarding * S. frutescens* and its cytotoxicological effects as a natural botanical drug utilising the zebrafish bioassay.

## Conclusion

We tested two kinds of extracts that are likely to be generated from *S. frutescens* and used in traditional medicine; and, commercialised as naturopathic tinctures. We confirmed the toxicity effects of each extract termed, S.fru-OH and S.fru-H_2_O, on the morphology, cardiotoxicity, mortality and hatching rate of zebrafish. The differences in the toxicity between S.fru-OH and S.fru-H_2_O prompted us to compare the profiles as different structures and properties of the compounds are likely extractable with use of two different solvents. The extracts had many compounds which are similar but we could separate the two using a metabolomic approach. This highlighted the relevance of sutherlandiosides as being important as differentiating chemical features for each respective plant-derived extract. Moreover, concentration effects linked to dosage may also cause different perceived toxicities in the zebrafish bioassay. In order to clarify the possible mechanism linked to the cytotoxic effects further analysis should be carried out. *S. frutescens* products are claimed to have immunomodulatory effects and consumers may take these as a dietary supplement to boost the immune system. However, consumption of the extracts as a daily tonic may have negative implications for human health and phytoextracts of this plant should thus be taken with care.

## Additional files

Additional file 1: **Figure S1.** Examples of the LC-MS spectra obtained with analysis of S.fru-H2O and S.fru-OH extract of *S. frutescens*. (PPTX 610 kb)

Additional file 2: **Figure S2.** Histogram of chemicals differentiating the ethanolic extract from the water extract linked to the S-plot presented in Fig. 5 (PPTX 63 kb)

## Abbreviations

dpf: days post fertilization
hpf: hours post fertilization
LC: Liquid chromatography
MS: Mass spectrometry
MTC: Maximum tolerable concentration
PLS-DA: Partial least squares-discriminant analysis
S.fru-H_2_O: *Sutherlandia* water extract
S.fru-OH: *Sutherlandia* ethanolic extract
