# Supplementary material for: Comparative cardio and developmental toxicity induced by the popular medicinal extract of Sutherlandia frutescens (L.) R.Br. detected using a zebrafish Tuebingen embryo model
Source: BMC Complement Altern Med. 2018 Oct 5;18:273. doi: 10.1186/s12906-018-2303-9 (PMC6173916; doi:10.1186/s12906-018-2303-9)
Supplement: Supplementary file 1 — Figure S1. Examples of the LC-MS spectra obtained with analysis of S.fru-H2O and S.fru-OH extract of S. frutescens. (PPTX 610 kb) [file 12906_2018_2303_MOESM1_ESM.pptx]

## Slide 1
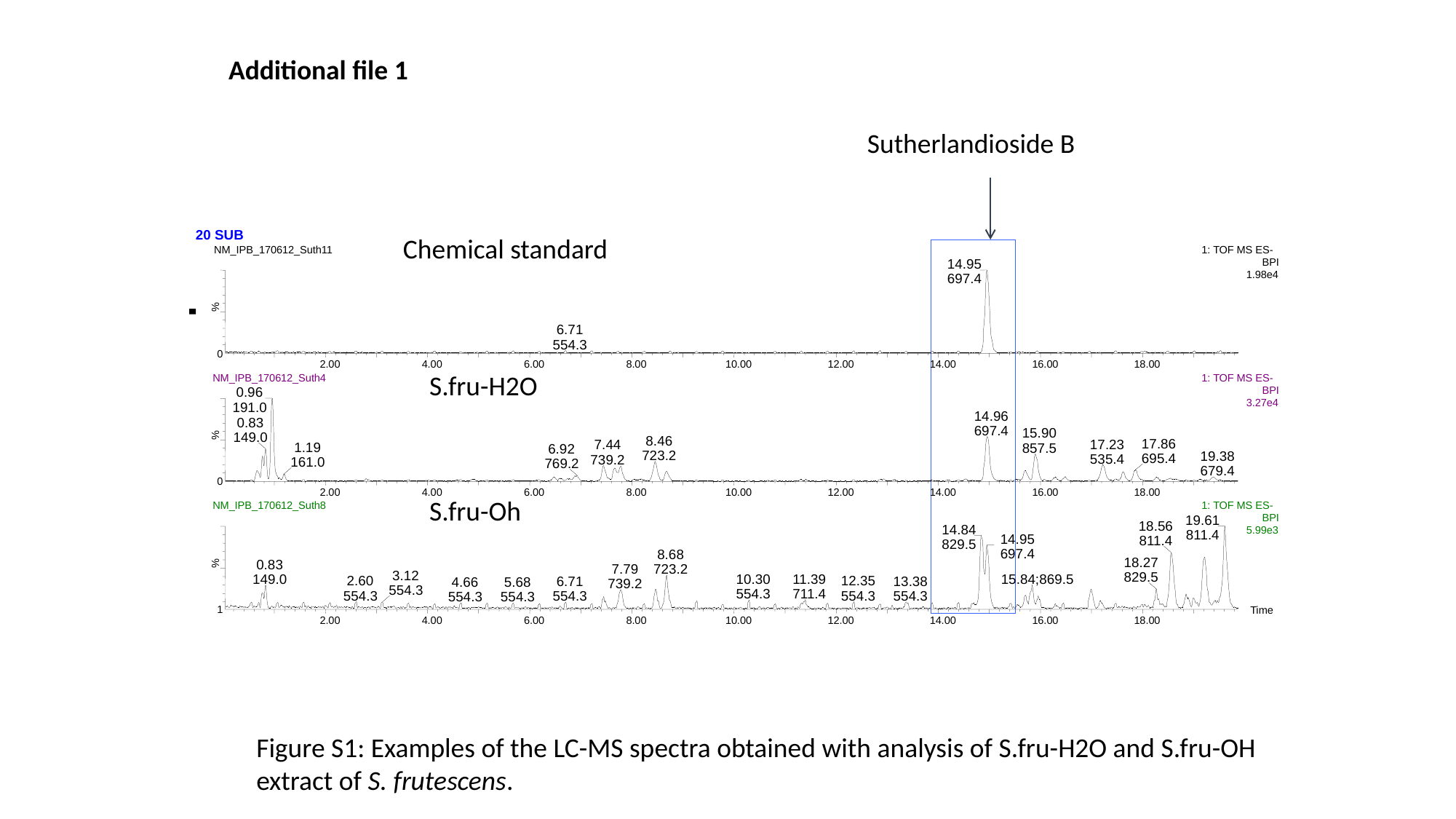

Additional file 1
Sutherlandioside B
20 SUB
%
1
Time
2.00
4.00
6.00
8.00
10.00
12.00
14.00
16.00
18.00
NM_IPB_170612_Suth11
1: TOF MS ES-
BPI
1.98e4
%
0
2.00
4.00
6.00
8.00
10.00
12.00
14.00
16.00
18.00
%
0
2.00
4.00
6.00
8.00
10.00
12.00
14.00
16.00
18.00
14.95
697.4
6.71
554.3
NM_IPB_170612_Suth4
1: TOF MS ES-
BPI
3.27e4
0.96
191.0
14.96
0.83
697.4
15.90
149.0
8.46
17.86
17.23
7.44
1.19
857.5
6.92
723.2
19.38
695.4
535.4
739.2
161.0
769.2
679.4
NM_IPB_170612_Suth8
1: TOF MS ES-
BPI
5.99e3
19.61
18.56
14.84
811.4
14.95
811.4
829.5
697.4
8.68
18.27
0.83
7.79
723.2
3.12
829.5
149.0
15.84;869.5
11.39
10.30
2.60
12.35
6.71
13.38
4.66
5.68
739.2
554.3
711.4
554.3
554.3
554.3
554.3
554.3
554.3
554.3
Chemical standard
S.fru-H2O
S.fru-Oh
Figure S1: Examples of the LC-MS spectra obtained with analysis of S.fru-H2O and S.fru-OH extract of S. frutescens.
