# Supplementary material for: Comparative cardio and developmental toxicity induced by the popular medicinal extract of Sutherlandia frutescens (L.) R.Br. detected using a zebrafish Tuebingen embryo model
Source: BMC Complement Altern Med. 2018 Oct 5;18:273. doi: 10.1186/s12906-018-2303-9 (PMC6173916; doi:10.1186/s12906-018-2303-9)
Supplement: Supplementary file 2 — Figure S2. Histogram of chemicals differentiating the ethanolic extract from the water extract linked to the S-plot presented in Fig. 5 (PPTX 63 kb) [file 12906_2018_2303_MOESM2_ESM.pptx]

## Slide 1
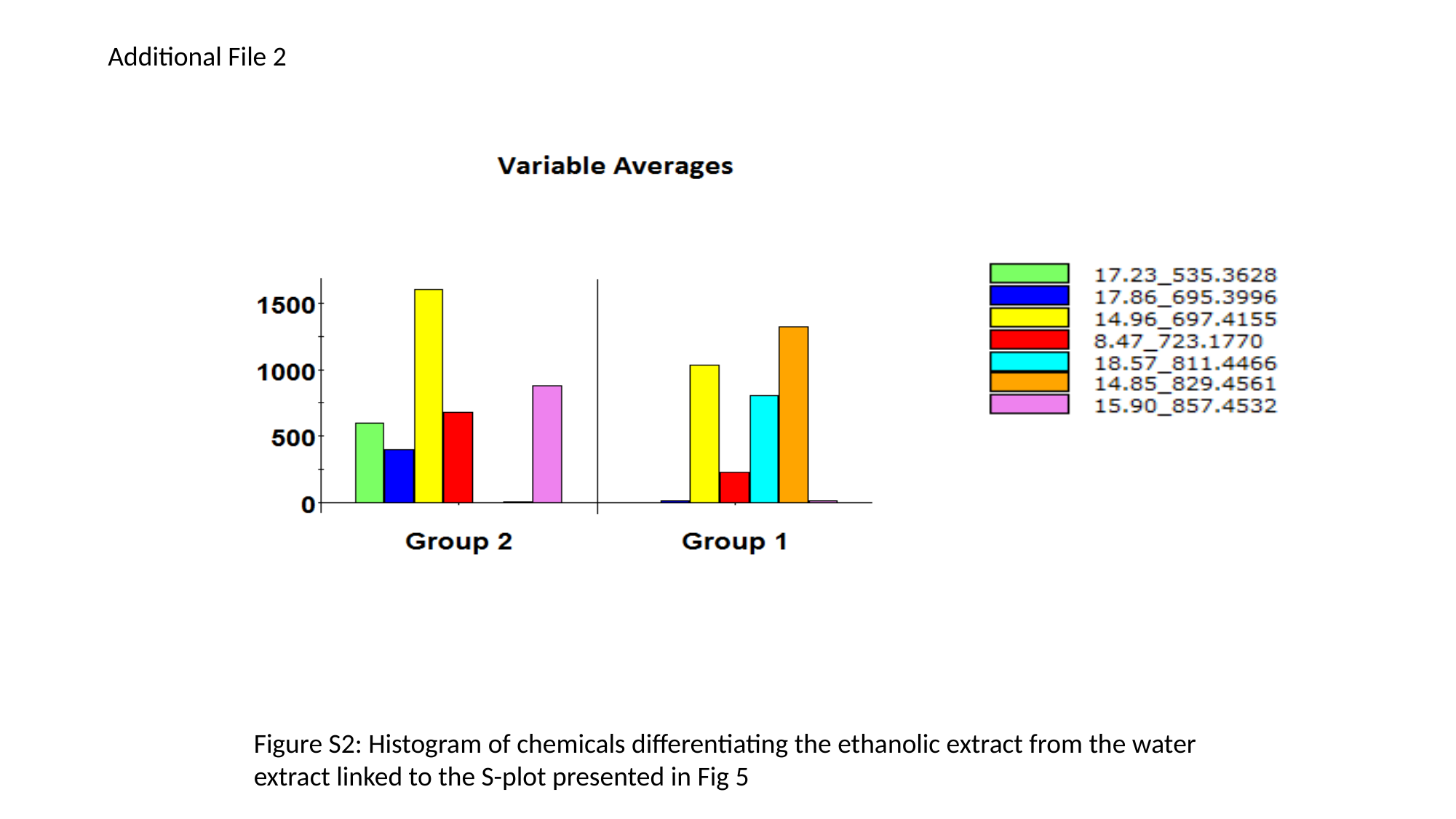

Additional File 2
Figure S2: Histogram of chemicals differentiating the ethanolic extract from the water extract linked to the S-plot presented in Fig 5
